# Classification of Heart Sounds Based on the Wavelet Fractal and Twin Support Vector Machine

**DOI:** 10.3390/e21050472

**Published:** 2019-05-06

**Authors:** Jinghui Li, Li Ke, Qiang Du

**Affiliations:** 1 Institute of Biomedical and Electromagnetic Engineering, Shenyang University of Technology, Shenyang 110870, China; 2 College of Telecommunication and Electronic Engineering, Qiqihar University, Qiqihar 161006, China

**Keywords:** heart sound, wavelet, energy entropy, fractal, twin support vector machine (TWSVM)

## Abstract

Heart is an important organ of human beings. As more and more heart diseases are caused by people’s living pressure or habits, the diagnosis and treatment of heart diseases also require technical improvement. In order to assist the heart diseases diagnosis, the heart sound signal is used to carry a large amount of cardiac state information, so that the heart sound signal processing can achieve the purpose of heart diseases diagnosis and treatment. In order to quickly and accurately judge the heart sound signal, the classification method based on Wavelet Fractal and twin support vector machine (TWSVM) is proposed in this paper. Firstly, the original heart sound signal is decomposed by wavelet transform, and the wavelet decomposition coefficients of the signal are extracted. Then the two-norm eigenvectors of the heart sound signal are obtained by solving the two-norm values of the decomposition coefficients. In order to express the feature information more abundantly, the energy entropy of the decomposed wavelet coefficients is calculated, and then the energy entropy characteristics of the signal are obtained. In addition, based on the fractal dimension, the complexity of the signal is quantitatively described. The box dimension of the heart sound signal is solved by the binary box dimension method. So its fractal dimension characteristics can be obtained. The above eigenvectors are synthesized as the eigenvectors of the heart sound signal. Finally, the twin support vector machine (TWSVM) is applied to classify the heart sound signals. The proposed algorithm is verified on the PhysioNet/CinC Challenge 2016 heart sound database. The experimental results show that this proposed algorithm based on twin support vector machine (TWSVM) is superior to the algorithm based on support vector machine (SVM) in classification accuracy and speed. The proposed algorithm achieves the best results with classification accuracy 90.4%, sensitivity 94.6%, specificity 85.5% and F1 Score 95.2%.

## 1. Introduction

Heart sound carries a lot of information about the health of cardiovascular system. It is an important source of information for diagnosing heart diseases and evaluating heart function. Heart sound contains a great deal of physiological and pathological information. The information comes from various parts of the heart, such as ventricle, atrium, great vessels, cardiovascular system and each valve. When the cardiovascular disease has not developed enough to produce clinical and pathological changes, murmurs and aberrations in heart sound are the important diagnostic information. In addition, heart sound has very important value in cardiovascular disease. It is an important non-invasive method for the detection of cardiovascular disease. It has irreplaceable advantages of electrocardiogram and ultrasound electrocardiogram. The phonocardiogram provides the visual heart sound waveform. Doctors can better understand and describe the patient’s heart problems by observing the patient’s phonocardiogram. It is helpful to overcome the inherent shortcomings of human ear auscultation and detect the abnormalities of heart sound in time by extracting the characteristics of heart sound signal making use of computer and analyzing them quantitatively. It is also helpful to realize the early and non-invasive screening of heart diseases [1].

Many processing and classification methods about heart sounds have been proposed such as wavelet transform (WT), hidden semi-Markov model (HSMM), logistic regression (LR), Mel-Frequency Cepstral Coefficients (MFCC), ensemble empirical mode decomposition (EEMD), deep neural network (DNN), deep convolutional neural network (CNN), Multi-fractal decomposition, Shannon energy and SVM [2,3,4,5,6,7,8,9,10]. The feature extraction methods were mainly based on these features, including short-time Fourier transform (STFT) features, kurtosis features, the wavelet features, deep structured features and the statistical features [3,5,11,12]. The classifiers also included such as hidden Markov model (HMM), neural network (NN), Linear Discriminate Analysis (LDA) and Naive Bayes [13,14,15]. The above methods had significant effects. Many methods were developed for more precise classification. But the running time is not an issue to be ignored. 

Under the premise of ensuring classification accuracy, in order to improve the running speed of this algorithm, the classification method based on wavelet fractal and twin support vector machine (TWSVM) is proposed. The method can better classify the normal and abnormal heart sound signals. It is beneficial to the judgment and diagnosis of heart diseases. In order to obtain more abundant feature information, the wavelet packet theory is used to solve its coefficient norm and energy entropy as feature vectors. This method can not only represent the abundant feature of heart sound, but also the dimension of the extracted feature vectors is not very large. Thus avoid the dimension disaster and reduce the running time. In addition, the heart sound signal is the deterministic and non-linear signal with obvious fractal characteristics. Based on the fractal theory, the fractal dimension of heart sound signal is calculated as the feature vector. The intrinsic characteristic of heart sound signal is revealed from the nonlinear perspective. It provides very important information for the feature of heart sound signal. Using the proposed feature extraction method, the implementation process is not complicated and the running time is also short.

The classifier uses Twin Support Vector Machines (TWSVM). It is based on support vector machine (SVM). Compared to SVM, TWSVM is looking for a pair of non-parallel hyper-planes, each of which should be as close as possible to one class of the samples and away from the other. The objective function of each quadratic programming corresponds to a specific class, and its constraints are affected by another class of samples. In the constraints of this quadratic programming problem, only positive or negative class of samples appears [16]. This characteristic coincides with the classification results of the heart sound signals. TWSVM is completely similar to SVM in form, but the algorithm ultimately comes down to solving two SVM-type problems. The computational overhead is reduced to 1/4 of the standard SVM. Twin Support Vector Machine (TWSVM) can effectively prevent the problem of samples imbalance. Therefore, this method is used to classify the heart sound signals, which achieves good classification results and saves a lot of running time.

The structure of this paper is organized as follows. The literature review about this proposed algorithm is presented in Section 2. Section 3 describes the algorithm implementation scheme and the correlation theory about the proposed heart sound algorithm. Section 4 introduces the algorithm implementation steps in detail. The partial procedure results are displayed. Section 5 shows the experimental results and discussion. This part introduces the experimental data and environment. The classification accuracy, running time, sensitivity, specificity and F1 Score about the proposed algorithm are given. The experimental results are compared with other algorithms. Finally, the main conclusions and future work are given in Section 6.

## 2. Literature Review

Many studies have already introduced the implement methods about heart sound classification. In this section, a review is briefly discussed about the previously existing approaches for heart sound signals. In 2009, Sepideh et al. presented a versatile multi-resolution algorithm based on the wavelet to extract the features. The statistical classifier and artificial neural network (ANN) were used alternatively to obtain the unique features. This experiment results showed that using Daubechies wavelet filter which was set according to this paper could obtain the best discrimination effects of the heart diseases in both classification approaches. Artificial Neural Network (ANN) brought more computational expense and time cost [17]. Samit Ari et.al proposed a compact and optimum design of neural network towards realtime detection of pathological patterns. Using the method of Singular Value Decomposition (SVD) selected the effective input features for the heart sound signal identification. The heart sound signal classification method used the overparameterized ANN structure. The best accuracy obtained was 99.279% [18]. In 2010, Avendano-Valencia et al. proposed the algorithm based on Parametric Time–Frequency Representations. The best accuracy obtained was 99.06 ± 0.06% [19]. Guraksin et al. proposed the heart sound signal classification method based on the least squares support vector machine in 2011. The wavelet Shannon entropy feature vectors were extracted. The least square support vector machine was used to classify these feature vectors. 96.6% of the classification performance was obtained [20]. In 2012, Cheng et al. proposed the algorithm based on a family of wavelets. The features of heart sounds were extracted by using of the heart sounds linear band frequency cepstral (HS-LBFC). The heart sound identification used the similarity distance method [21]. In 2014, Patidar et al. presented the method of using constrained tunable-Q wavelet transform to classify the cardiac sound signals. The classification method used the least squares support vector machine (LS-SVM) with various kernel functions. The best accuracy obtained was 94.01% [22]. In 2016, Abo-Zahhad et al. proposed the new method based on wavelet packet cepstral features. The proposed features used the non-linear wavelet packet filter banks. They were set to fit the acoustic nature of the heart sound. Use the database HSCT-11 to evaluate the proposed system. From the obtained results, the best identification accuracy about the proposed system was 91.05% [23]. In 2017, Zhang et al. proposed the classification method based on scaled spectrogram and tensor decomposition. The spectrograms of the detected heart cycles were firstly scaled to a fixed size. The most discriminative features were extracted by the dimension reduction process of the scaled spectrograms. The features of the scaled spectrograms were extracted by using of the tensor decomposition method. The proposed algorithm was performed on the datasets which were supported by 2016 PhysioNet challenge and the PASCAL classifying heart sounds challenge. The highest normal precision was 96% [24]. In 2018, Maryam Hamidi et al. presented the heart sound classification method based on curve fitting and fractal dimension. The information contained in the heart sound signal was obtained by curve fitting. The useful features which were extracted by MFCC2 were fused with the fractal features by stacking. The classification method used the nearest neighbor classifier. The proposed method was performed on the datasets which were used for competition in 2016. By experiments, the overall accuracy of 81%, 92% and 98% on the three datasets were respectively achieved [13]. Yaseen et al. proposed the heart sound classification method based on multiple features. The Discrete Wavelets Transform (DWT) and Mel Frequency Cepstral Coefficient (MFCCs) were used to extract the characteristics of heart sound signals. The deep neural network (DNN), support vector machine (SVM) and centroid displacement based *k* nearest neighbor was used for classification. The MFCCs and DWT features were combined to improve the classification accuracy. The classification used SVM and DWT. The proposed method was used to diagnose the heart diseases. It could obtain the 97% accuracy [25]. In the following section, our proposed feature extraction and classification algorithm is discussed in details. 

## 3. Methodology

**Figure 1:**
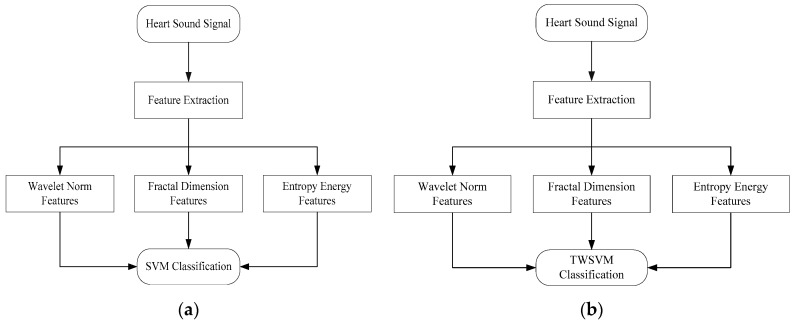
The steps to implement this algorithm: (**a**) Proposed algorithm using SVM; (**b**) Proposed algorithm using TWSVM.

Heart sounds contain abundant information, which can reflect the state of heart and cardiovascular operation. Thus reflect the pathological changes in the heart. The value of heart sound signals depends on the ability of extracting pathological information. Extracting the corresponding features from heart sound signals can provide the auxiliary basis for the diagnosis and treatment of heart diseases. So a lot of signal processing methods can be performed on the heart sound signals. The processing steps of heart sound signal are, the heart sound signal acquisition, noise removal, the heart sound signal sampling, feature extraction, and signal classification. In this proposed algorithm, the heart sound signal acquisition, noise removal and the heart sound signal sampling have been firstly finished. Feature extraction and signal classification are the cores of the working in this paper. According to the proposed algorithm, the steps to implement this algorithm are shown in the following Figure 1.

### 3.1. Wavelet Packet Theory

Since the wavelet transform only further decomposes the low frequency part of the signal, the high frequency part, that is, the detail part of the signal, does not continue to decompose. Unlike the wavelet transform, the high frequency part can be decomposed more precisely by wavelet packet transform. This decomposition is neither redundant nor omitted, so it can perform better time-frequency localization analysis of the signal. Therefore, this paper uses the method of wavelet packet decomposition to extract some features of the heart sound signal.

#### 3.1.1. Definition of Wavelet Packet

The so-called orthogonal wavelet packet, roughly speaking, is a family of functions. The standard orthogonal bases of $L^{2}(R)$ can be constructed from them. Here, many groups of standard orthogonal bases of $L^{2}(R)$ can be selected. The Orthogonal wavelet base is usually one of them. The wavelet function is one of the wavelet packet functions family. So the wavelet packet is the generalization of wavelet function.

The orthogonal low-pass real coefficient filter is ${\left\{h_{k}\right\}}_{k\in Z}$ corresponding to the orthogonal scaling function ϕ(t). The high-pass filter is ${\left\{g_{k}\right\}}_{k\in Z}$ corresponding to the orthogonal wavelet function ψ(t), where $g_{k}={\left(-1\right)}^{k}h_{1-k}$. They satisfy the following scale equation and wavelet equation:

(1) $$\left\{ \begin{array}{l} \phi (t)=\sqrt{2}\sum_{k\in Z}h_{k}\phi (2t-k)\\ \psi (t)=\sqrt{2}\sum_{k\in Z}g_{k}\phi (2t-k) \end{array}\right.$$

In order to express the wavelet packet function conveniently, the following new notation is introduced:

(2) $$\left\{ \begin{array}{l} \mu_{0}(t):=\phi (t)\\ \mu_{1}(t):=\psi (t) \end{array}\right.$$

Equation (2) can be expressed as:

(3) $$\left\{ \begin{array}{l} \mu_{0}(t)=\sqrt{2}\sum_{k\in Z}h_{k}\mu_{0}(2t-k)\\ \mu_{1}(t)=\sqrt{2}\sum_{k\in Z}g_{k}\mu_{0}(2t-k) \end{array}\right.$$

A set of functions called wavelet packet can be defined by $\mu_{0}$, $\mu_{1}$, h, g on the fixed scale.

(4) $$\left\{ \begin{array}{l} \mu_{2n}(t)=\sqrt{2}\sum_{k}h_{k}\mu_{n}(2t-k)\\ \mu_{2n+1}(t)=\sqrt{2}\sum_{k}g_{k}\mu_{n}(2t-k) \end{array}\right.$$

Function $\mu_{n},n=0,1,2,L$ defined recursively by Equation (4) is called wavelet packet which is determined by orthogonal scaling function $\mu_{0}=\phi$ [26,27,28].

**Figure 2:**
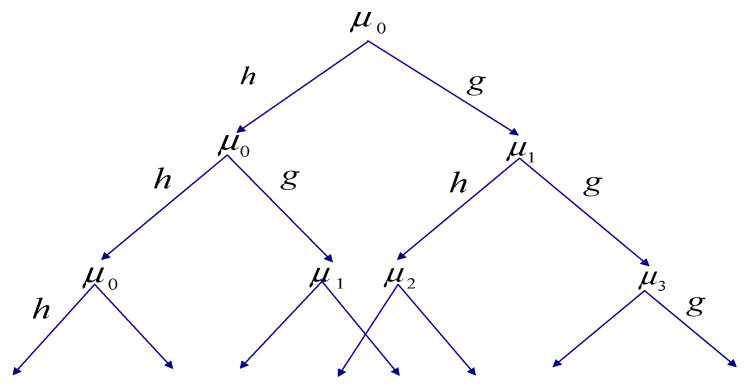
The wavelet packet under the fixed scale.

The wavelet packet can be represented by a complete binary tree, as shown in Figure 2.

#### 3.1.2. Wavelet Packet Basis Function

This paper uses the Daubechies wavelet, referred to as *db* wavelet. The order is *N* in *dbN*, *N* = 2 ~ 10. When *N* = 1, the *db1* is Haar wavelet. The *db* wavelet has both orthogonality and biorthogonality. It is tightly supported. Except for *N* = 1, the *dbN* is not symmetric and has no explicit expression. The *db* wavelet is a typical orthogonal wavelet, which is widely used.

#### 3.1.3. Wavelet Packet Energy Entropy

Entropy can be used to measure the uncertainty of information contained in one-dimensional signal or two-dimensional image. The energy entropy of the wavelet packet obtained here has the characteristics of both feature representation and feature dimension reduction. Its theory is as follows:

Assuming that the length of the sample signal to be analyzed is *N*, the signal is decomposed by wavelet packet and the decomposition level is *j*. Then the decomposition coefficients are reconstructed and the sequence of the reconstructed coefficients is expressed as follows: $S_{jk},(k=0∼2^{j}-1)$. Let $E_{jk}={\left|S_{jk}(i)\right|}^{2}$, $E_{jk}$ represents the power value of the *k*-th node distribution of the reconstructed sequence on the *j*-th decomposition level. Let $\varepsilon_{jk}(i)=E_{jk}/E$, where

(5) $$E=\left[\sum_{k=0}^{2^{j}-1}{\left|E_{jk}\right|}^{1/2}\right]$$

Therefore

(6) $$\sum_{k}\varepsilon_{jk}=1$$

The energy entropy measure of wavelet packet is defined as

(7) $$H_{jk}=-\sum_{i=1}^{N}\varepsilon_{jk}(i)\log\left|\varepsilon_{jk}(i)\right|$$

In the Equation (7), $H_{jk}$ represents the energy entropy of the *k*-th wavelet packet of the signal on the *j*-th decomposition layer [11,20].

### 3.2. Fractal Theory

Fractal dimension is an important parameter to quantitatively describe the irregularity and complexity of fractal set in fractal theory. The size of fractal dimension reflects the complexity, fineness, irregularity and space-filled degree of contour in space. The bigger the fractal dimension is, the richer the details are. The smaller the fractal dimension is, the less the details are. There are many methods to calculate fractal dimension: differential box counting method, carpet covering method, power spectrum method, mesh cell counting method and probability estimation. In practice, box dimension method has been widely used because of its simple calculation and easy empirical estimation. In this paper, we use the binary box dimension estimation method to extract the fractal dimension features of heart sound signals, which provide important information for the characteristics of heart sound signals. This paper mainly introduces this method.

The binary box dimension estimation method is the fractal dimension estimation method derived from the definition of box dimension. According to the definition of box dimension, in space $R^{2}$, box dimension $D_{B}$ can be defined as:

(8) $$D_{B}=\underset{\delta \to 0}{\lim}\frac{\log N_{\delta }(F)}{\log(1/\delta )}$$

Here, $N_{\delta }(F)$ represents the minimum number of meshes of covering F by a square with sides that are δ in length.

According to Equation (8), the minimum number of meshes $N_{\delta }(F)$ of covering F obeys the power law, that is:

(9) $$N_{\delta }(F)∼\delta^{-D_{B}}$$

Take the logarithm of both sides of Equation (9):

(10) $$\log\;N_{\delta }(F)∼D_{B}\;\log(1/\delta )$$

Equation (10) shows that the asymptote of the curve $\log N_{\delta }(F)$—log(1/δ) is a straight line whose slope is $D_{B}$ when δ→0.

In practical applications, the time series of the signal is generally composed of discrete points. When calculating its fractal dimension, the grid scale δ cannot be reduced to zero indefinitely. The sampling period of the signal must be fully considered. In addition, the fractal characteristics of the signals are generally self-similarity in statistical sense. Therefore, $D_{B}$ only needs to be estimated within a certain grid scale. That is to say, in a suitable scale range, the grid scales are changed according to certain rules, and the corresponding $N_{\delta }(F)$ values are calculated according to the different δ values. Then the least square method is used to linearly fit the set which is constructed by the points (log(1/δ), $\log N_{\delta }(F)$). The slope of the fitting line is the estimated value of $D_{B}$. It can be seen that when estimating box dimension $D_{B}$, the range and variation of grid scale have the important influences on the accuracy and the speed of estimating [29,30,31].

## 4. The Proposed Algorithm Based on Wavelet Fractal and TWSVM

Aiming at the classification problem of heart sound signals, this paper proposes the algorithm based on wavelet fractal and twin support vector machine (TWSVM) to classify the heart sound signals which are selected from the PhysioNet/CinC Challenge 2016 heart sound database [32,33]. Using the algorithm achieves the good classification results. The running speed is also greatly improved compared to the algorithm based on support vector machine (SVM). The implementation steps of this algorithm are described below.

**Figure 3:**
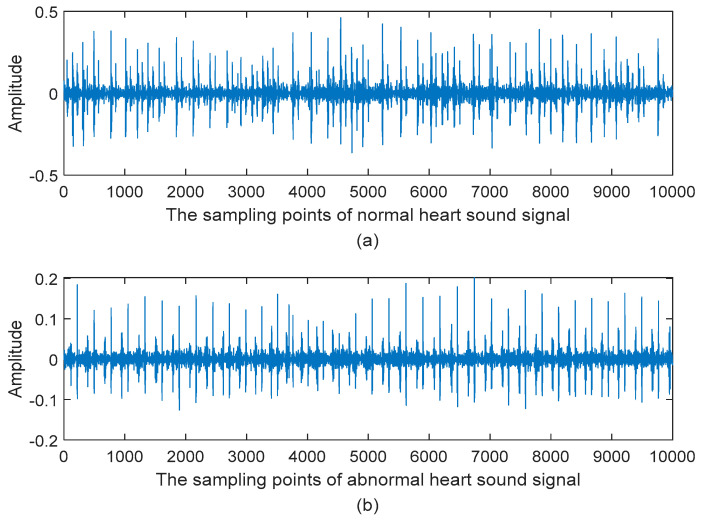
The waveforms of heart sound signals: (**a**) Normal heart sound signal; (**b**) Abnormal heart sound signal.

The implementation steps of this algorithm are as follows:

Step 1: Read the heart sound signals from the database, and divide the heart sound signals into two parts: training set and testing set. The following Figure 3 is the waveforms of heart sound signals from the database. The 10,000 sampling points are taken in Figure 3 and the sampling frequency is 2000 Hz. The abscissa is the sampling points and the ordinate is the amplitude of heart sound signal. Figure 3a shows the waveform of the normal heart sound signal and Figure 3b shows the waveform of the abnormal heart sound signal.

Step 2: Feature extraction. The heart sound signal is decomposed into four-layer wavelet packet using the Shannon entropy and the *db6* wavelet of Daubechies wavelet family. Then the decomposition coefficient tree is generated. Select the wavelet packet decomposition coefficients of 16 nodes about (4,0), (4,1), (4,2), (4,3), (4,4), (4,5), (4,6), (4,7), (4,8), (4,9), (4,10), (4,11), (4,12), (4,13), (4,14) and (4,15), which reflect both low-frequency approximation information and high-frequency detail information of the signal. A total of 16 coefficient matrices are obtained. Then two-norm values of these coefficient matrices are calculated. The obtained data compose the partial feature vectors of heart sound signals.

**Figure 4:**
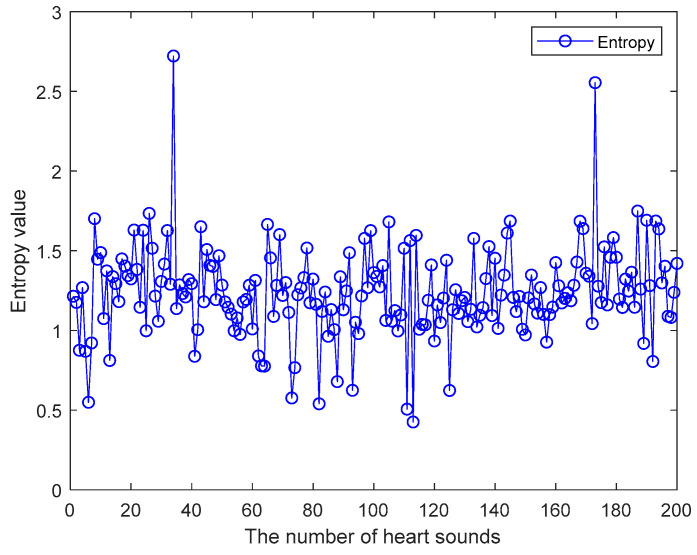
The wavelet energy entropy eigenvalues.

Step 3: Calculate the wavelet packet energy entropy characteristics of heart sound signals. For the above four-layer wavelet packet decomposition of heart sound signal, 16 nodes of coefficient matrices have been obtained. So the sum of the energies of the coefficient matrices will be calculated. Then the wavelet energy entropy of heart sound signal can be calculated by using the definition of wavelet packet energy entropy. Figure 4 shows the wavelet energy entropy eigenvalues. These heart sound signals come from the PhysioNet/CinC Challenge 2016 heart sound database [32,33], which are selected randomly. A total of heart sound signals are 200. The wavelet energy entropy eigenvalues mostly fluctuate between 0.5 and 2.

**Figure 5:**
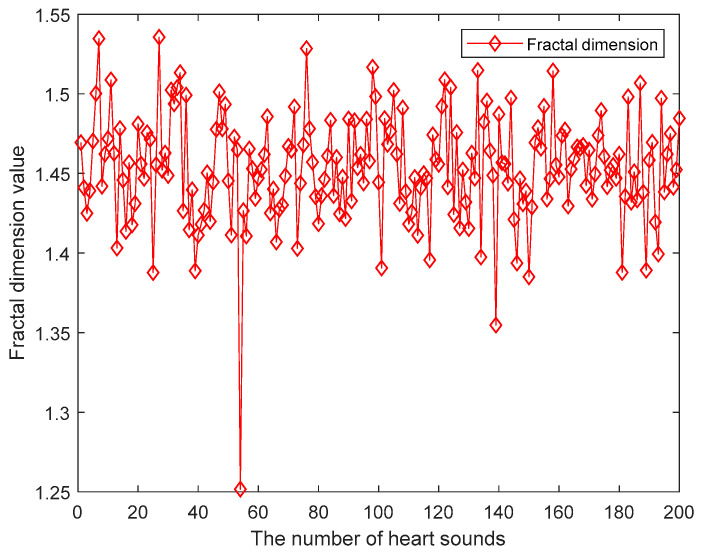
The fractal dimension eigenvalues.

Step 4: Use the binary box dimension estimation method to obtain the fractal dimension characteristics of the heart sound signals. The grid scale $\delta_{i}$ is changed according to the binary increment, namely $\delta_{i}=2^{i-1}\delta_{t},i=1,2,\ldots ,i_{\max}$, $\delta_{t}$ is the time interval between two adjacent sample points of heart sound signal. The estimated value of the box dimension $D_{B}$ is calculated by the estimation method. Thereby obtain the fractal dimension eigenvalues of the heart sound signals. Figure 5 is the fractal dimension eigenvalues distribution diagram. The heart sound signals are the same as the above. From the graph, the fractal dimension eigenvalues change between 1.35 and 1.55. But there are still special points not in the range.

**Figure 6:**
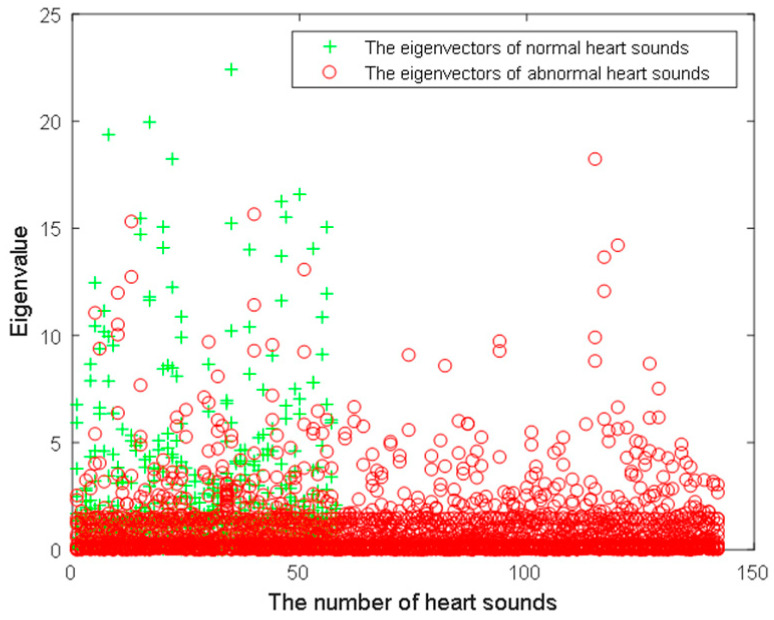
The heart sound signal eigenvectors distribution.

Step 5: Combine the above 2 norm eigenvalues of the wavelet packet coefficients, the wavelet energy entropy eigenvalues and the fractal dimension eigenvalues as the eigenvectors of the heart sound signals. And input them to the twin support vector machine (TWSVM) for training and testing. Figure 6 shows the scatter plot of the eigenvectors. The red points represent the eigenvectors of abnormal heart sound signals. And the green points represent the eigenvectors of normal heart sound signals. The number of heart sound signals selected is still 200 like the above, including 142 abnormal heart sound signals and 58 normal heart sound signals. From the graph, it can be reflected that the eigenvectors distribution is dense. How to classify these data correctly is the key to choose the appropriate classifier. Here, the twin support vector machine (TWSVM) is used to classify. It uses the kernel function to map low-dimensional spatially inseparable samples into high-dimensional space, making these samples divisible in high-dimensional space. It can solve the classification problem of two categories and the imbalance problem of samples very well.

Step 6: Use the twin support vector machine (TWSVM) to classify the heart sound signals. The twin support vector machine (TWSVM) can classify the heart sound signal into two categories: normal and abnormal. Because the original thought of the twin support vector machine (TWSVM) algorithm is the classification problem of two categories. Unlike traditional support vector machine (SVM) which only looks for a hyper-plane, the twin support vector machine (TWSVM) looks for two non-parallel hyper-planes. In this paper, the classification of heart sound signals belongs to the non-linear separable classification problem. TWSVM combines with the kernel technology to deal with it. 

The basic idea is as follows: firstly, the data in the original feature space is mapped into the high-dimensional regenerative space, so that the mapped data samples can be linearly separable; secondly, two hyper-planes of TWSVM are established in the high-dimensional space. The K(x,y) stands for kernel function. Suppose there are m training samples in $R^{n}$ space. They all have n attributes. Among them, $m_{1}$ samples belong to positive class and $m_{2}$ samples belong to negative class. They are represented by matrix A and matrix B respectively. Each row of matrix A represents a sample point belonging to positive class, and each row of matrix B represents a sample point belonging to negative class. For non-linear classification problems about two categories, TWSVM constructs the following two hyper-planes based on kernel functions:

$$K(x^{T},C^{T})u_{1}+b_{1}=0\;\mathrm{and}\;K(x^{T},C^{T})u_{2}+b_{2}=0$$

Where, $u_{1}$ and $u_{2}$ are the normal vectors of two hyper-planes, $b_{1}$ and $b_{2}$ is the offset of two hyper-planes. The following quadratic programming problem is constructed and solved to obtain the normal vectors and the offsets of two non-parallel hyper-planes:

(11) $$\begin{array}{l} (\mathrm{TWSVM}1)\;\min\frac{1}{2}{\left\|K(A,C^{T})u_{1}+e_{1}b_{1}\right\|}^{2}+c_{1}e_{2}^{T}\zeta \\ subject\;to-(K(B,C^{T})u_{1}+e_{2}b_{1})+\zeta \geq e_{2},\zeta \geq 0 \end{array}\}$$

(12) $$\begin{array}{l} \begin{array}{l} (\mathrm{TWSVM}2)\;\min\frac{1}{2}{\left\|K(B,C^{T})u_{2}+e_{2}b_{2}\right\|}^{2}+c_{2}e_{1}^{T}\eta \\ subject\;to\;(K(A,C^{T})u_{2}+e_{1}b_{2})+\eta \geq e_{1},\eta \geq 0 \end{array}\} \end{array}$$

Where, matrix C represents all training samples, and each row of matrix C is a sample of training. The penalty parameters are $c_{1}$ and $c_{2}$. The $e_{1}$ and $e_{2}$ are column vectors whose elements are 1. The slack variables are ζ and η. The kernel function K(x,y) can map the finite dimensional data to high dimensional space, making these data linear separable in high dimensional space. Two kernel-based hyper-planes of TWSVM are obtained by solving Equations (11) and (12). In the case of nonlinearity, the decision rules of TWSVM can be summed up as the test sample belonging to the class which hyper-plane is close to it. Each of the hyper-planes is very close to one class and as far as possible from the other. The specific decision function is as follows:

(13) $$\mathrm{Label}(x)=\underset{k=1,2,\ldots ,K}{\arg\;\min}\left(K(x,C^{T})u_{k}+b_{k}/\sqrt{u_{k}^{T}K(C,C^{T})u_{k}}\right)$$

For a new sample $x_{0}$, calculate its vertical distance to the two hyper-planes. If its distance to the positive hyper-plane is less than its distance from the negative hyper-plane, it belongs to the positive class. Otherwise it belongs to the negative class [34,35,36,37,38]. Based on the above principle, the classifier can be used to quickly and effectively divide the heart sound signals into two categories: normal and abnormal.

## 5. Experimental Results and Discussion

### 5.1. Experimental Data

For the proposed algorithm, PhysioNet/CinC Challenge 2016 heart sound database is used to verify the algorithm. The database contains normal heart sound signals collected from healthy people and abnormal heart sound signals collected from patients with certain aspects of heart disease. The database contains more than 2000 records of normal and abnormal heart sound signals [32,33,39]. In this paper, a sub-database from PhysioNet/CinC Challenge 2016 heart sound database is selected for this experiment. The 200 heart sound signal records are randomly selected from the database as the training set. The 150 heart sound signal records are selected as the testing set.

### 5.2. Experimental Environment

The computer processor used in this experiment is Intel (R) Celeron (R) CPU N3450 1.10 GHz. RAM is 4.00 GB. The system is 64-bit Windows 10 operating system. The simulation software is MATLAB 2018b.

### 5.3. Comparison of Experimental Results and Analysis

The traditional SVM classification method and the proposed method based on the twin support vector machine (TWSVM) are compared in terms of running time and classification accuracy. In addition, the experimental results are also compared with other literatures [11,40,41]. In order to highlight the superiority of the proposed algorithm, in the experimental process, the extracted features are compared in three forms, and the classification results are compared by SVM and TWSVM. The first feature extraction method only extracts the two-norm eigenvectors of the wavelet packet coefficients, and each signal extracts 16-dimensional eigenvectors. The second feature extraction method adds the wavelet energy entropy feature based on the first extraction method. The feature dimension is a total of 17-dimensional eigenvectors. The third feature extraction method is based on the second extraction method, which adds the fractal dimension feature of the heart sound signal. The obtained feature dimension is a total of 18-dimensional eigenvectors. 

In this paper, the classification accuracy, specificity, sensitivity and F1 Score are used to evaluate the performance of the proposed feature extraction and classification algorithm. The classification accuracy is namely identification accuracy. These performance parameters are calculated according to Equations (14)–(18). They are described as follows: *TP*, true positive number, which represents that some abnormal subjects are correctly identified as abnormal subjects; *FN*, false negative number, which represents that some people with abnormal heart sounds are identified as healthy people; *TN*, true negative number, which represents that some healthy people are correctly identified as healthy people; and *FP*, false positive number, which represents that some healthy people are identified as having abnormal heart sounds [22,31,42]. However, the classification results also depend on the methods of feature extraction for the application.

(14) $$Accuracy=\frac{TP+TN}{TP+FP+FN+TN}\times 100\%$$

(15) $$Sensitivity=Recall=\frac{TP}{TP+FN}\times 100\%$$

(16) $$Specificity=\frac{TN}{FP+TN}\times 100\%$$

(17) $$Precision=\frac{TP}{TP+FP}\times 100\%$$

(18) $$F1\;Score=\frac{2\times Recall\times Precision}{Recall+Precision}\times 100\%$$

The following is the comparison results of classification using SVM and TWSVM based on the three kinds of feature extraction methods: (1) Wavelet; (2) Wavelet + Entropy; (3) Wavelet + Entropy + Fractal. As shown in Table 1, Table 2 and Table 3, the classification accuracy and running time are compared, which are obtained in the testing process.

**Figure 7:**
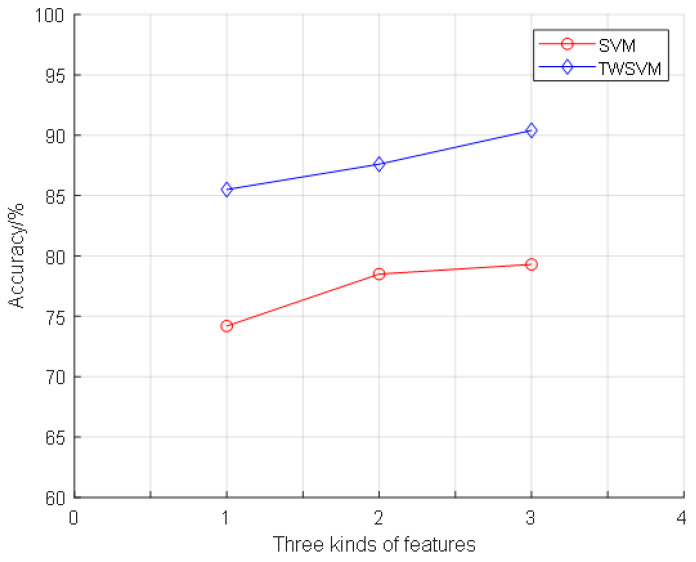
The comparison of classification accuracy.

**Figure 8:**
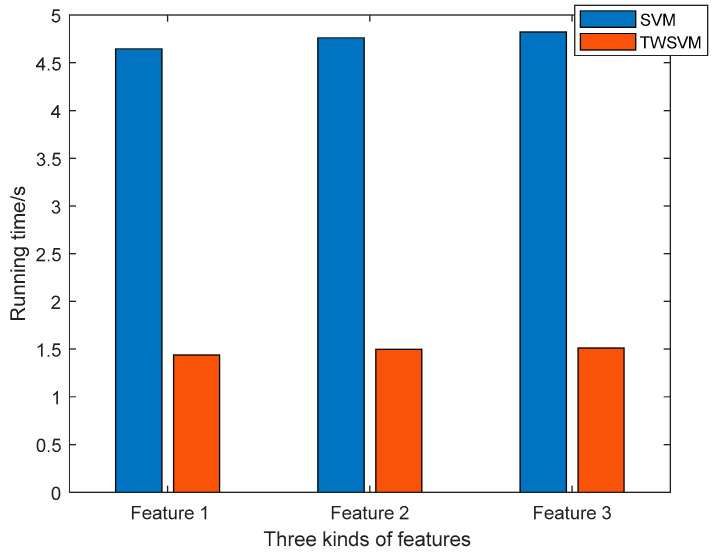
The comparison of running time.

In order to display the comparison results more intuitively, the results are drawn and compared. As shown in Figure 7 and Figure 8, it can be clearly seen that the proposed algorithm is superior to the SVM method in terms of classification accuracy and running time. The TWSWM classification method can better reflect the advantages of time cost. The third feature extraction method obtains the best classification accuracy.

For the parameter setting of twin support vector (TWSVM), the Gaussian kernel $K({x,y)=e}^{-\frac{{\left\|x-y\right\|}^{2}}{2\sigma^{2}}}$ is selected as the kernel function, where σ is the kernel parameter [38,43]. It controls the radial range of action. In order to estimate the classification accuracy of the proposed algorithm, the 10-fold cross validation method is used in the experiment. The optimal parameters of the algorithm are determined on the basis of obtaining the best classification accuracy through multiple 10-fold cross validations. For TWSVM, two hyper-planes need to be determined. The parameters to be determined are penalty parameters $c_{1}$, $c_{2}$ and kernel parameters $\sigma_{1}$, $\sigma_{2}$. Through continuous experiments and accumulated experience, it is found that the best classification accuracy is obtained when $c_{1}$, $c_{2}$, $\sigma_{1}$, $\sigma_{2}$ are all selected as 3.5. However, for twin support vector machine (TWSVM), how to select parameters is still a problem in exploration.

In addition, we also use sensitivity, specificity and F1 Score besides classification accuracy and running time to evaluate the performance of feature extraction and classification methods. Table 4 shows the comparison results of three kinds of feature extraction methods using SVM and TWSVM respectively. From the table, the proposed method obtains the best performance results with 90.4%, 94.6%, 85.5% and 95.2% in terms of classification accuracy, sensitivity, specificity and F1 Score respectively. The proposed algorithm based on TWSVM is superior to this algorithm based on SVM.

Compared with other literatures [11,40,41], the proposed method in this paper reduces the dimension of feature vectors, which saves a lot of running time in the dimension processing. The Table 5 shows the comparison results of the literatures and the proposed algorithm. It can be seen from the table that the proposed algorithm reduces the feature dimension without reducing the classification accuracy. Using the TWSVM classification method makes the overall running time of the algorithm reduced greatly. It lays the theoretical foundation for the further practical development of this algorithm.

## 6. Conclusions

In this paper, the heart sound signal classification method based on Wavelet Fractal and twin support vector machine (TWSVM) is proposed. The method extracts the node coefficient matrix by wavelet packet decomposition and calculates its two-norm values to obtain the partial eigenvectors of the heart sound signal. In order to characterize the heart sound signal more accurately, the energy entropy and fractal dimension characteristics of heart sound signal are extracted by using wavelet packet energy entropy theory and fractal dimension theory. In the operation of the algorithm, it is more time-saving and more complete to express the signal feature than other feature extraction methods. Finally, the efficient twin support vector machine (TWSVM) classifier is used to classify the normal and abnormal heart sound signals. It obtains the good classification results.

Although the proposed algorithm achieves good results in both running time and classification accuracy, the classifier needs to be improved further in order to obtain the better classification accuracy. The twin support vector machine (TWSVM) should be developed in the future research work so as to be able to identify the categories of disease signals which belong to the abnormal signals. In this way, the improvement of TWSVM can realize the judgment of many heart diseases, and then it can be applied to the diagnosis and treatment of clinical heart diseases in the future.

## Figures and Tables

**Table 1:** The classification results based on the wavelet.

Classifier	Features	Accuracy	Running Time
SVM	Wavelet	74.2%	4.647s
TWSVM	Wavelet	85.5%	1.438s

**Table 2:** The classification results based on the wavelet and energy entropy.

Classifier	Features	Accuracy	Running Time
SVM	Wavelet + Entropy	78.5%	4.762s
TWSVM	Wavelet + Entropy	87.6%	1.499s

**Table 3:** The classification results based on the wavelet, energy entropy and fractal dimension.

Classifier	Features	Accuracy	Running Time
SVM	Wavelet + Entropy + Fractal	79.3%	4.822s
TWSVM	Wavelet + Entropy + Fractal	90.4%	1.512s

**Table 4:** Comparison of Accuracy, sensitivity, specificity and F1 Score about three kinds of feature extraction methods based on SVM and TWSVM.

Classifiers	Features	Sensitivity	Specificity	Accuracy	F1 Score
SVM	Wavelet	81.4%	66.7%	74.2%	82.2%
	Wavelet + Entropy	85.2%	71.2%	78.5%	85.6%
	Wavelet + Entropy + Fractal	86.3%	73.5%	79.3%	86.9%
TWSVM	Wavelet	88.5%	81.5%	85.5%	89.2%
	Wavelet + Entropy	90.3%	83.8%	87.6%	90.6%
	Wavelet + Entropy + Fractal	94.6%	85.5%	90.4%	95.2%

**Table 5:** The comparison of the proposed algorithm and the literatures.

Feature Extraction Methods	Feature Dimension	Sensitivity	Specificity	Accuracy
OMS-WPD [11]	27	85.29%	94%	88.98%
DFT/Burg AR-PCA-ANN [41]	33	97.44%	90.48%	95%
DFT/ANN [40]	300	97.29%	82.6%	91.67%
Proposed algorithm	18	94.6%	85.5%	90.4%
